# Symmetric peripheral gangrene: Catch it early!

**DOI:** 10.4103/0974-2700.62119

**Published:** 2010

**Authors:** Swagata Tripathy, Biswajeet Rath

**Affiliations:** Department of Anaesthesia and Intensive Care, Bhubaneswar, India; 1 Cardiology, Kalinga Institute of Medical Sciences, Bhubaneswar, India

## CASE REPORT

A 75-year-old female presented to us with history of fever, diarrhea and vomiting for 4 days after being referred from a nursing home. On arrival, she was disoriented and dehydrated. Her heart rate was 110/min, blood pressure 70/30 mmHg and respiratory rate 30/min. Investigations revealed elevated total cell counts and deranged renal and liver function tests. A diagnosis of viral gastroenteritis and septic shock was made. Fluid resuscitation and elective ventilation were started followed by noradrenaline infusion at 1.5 U/min (through the subclavian vein). There was no history of jaundice, hematuria, Raynaud's phenomenon, joint pains, taking B-blockers, ergot etc.

**Figure 1:**
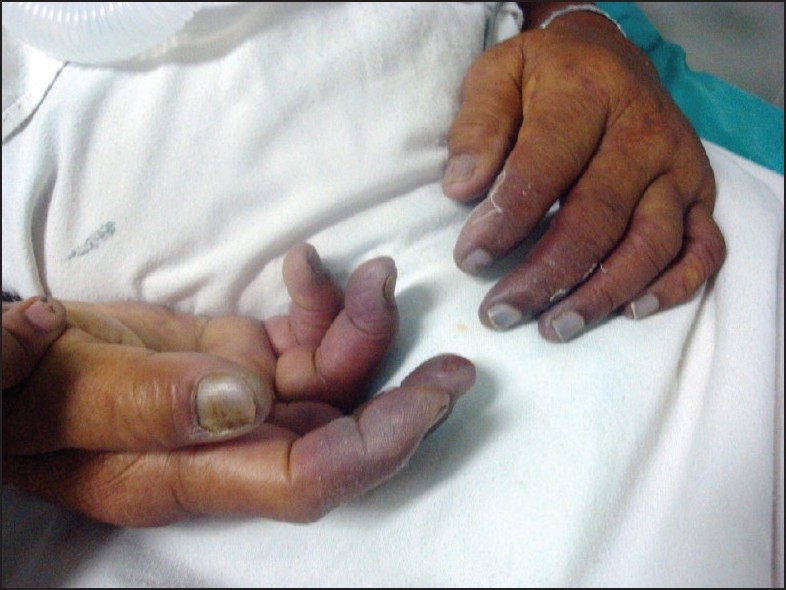
Initiation of cyanosis in fingertips on (Day 2)

**Figure 2:**
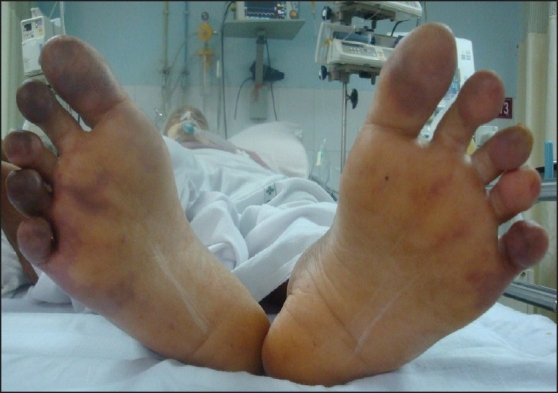
Bilateral toes showing cyanosis on (Day 2)

On the second day, cyanosis was observed in the fingers, palms and toe tips with, finger tips being more visibly affected [Figures 1 and 2]. Radial and dorsalis pedis pulsations were well felt in all limbs. An immediate suspicion of disseminated intravascular coagulation (DIC), warming of extremities, adding linezolid for possible gram positive infection, prompt attempts at withdrawal of noradrenalin and starting therapeutic dose of enoxaparin and fresh frozen plasma infusion ensued. Investigations like prothrombin time 28 s (International normalized ratio 2.8), platelets 70,000/cmm, fibrin degradation products positive, hematuria, oliguria and acute renal failure confirmed DIC.

**Figure 3:**
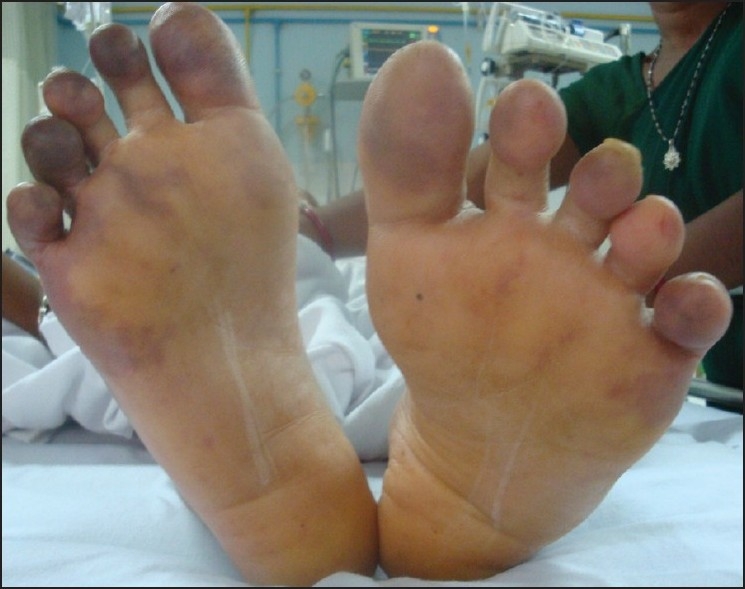
Improvement observed on left toes (Day 5)

**Figure 4:**
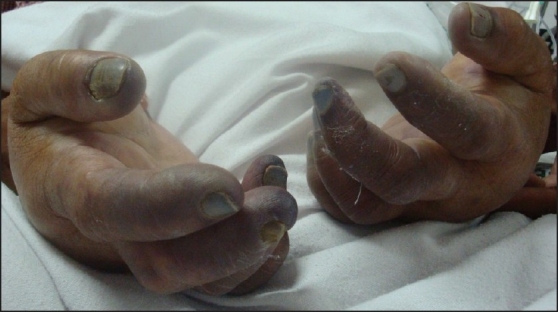
Established gangrene in fingers (Day 7)

The patient's general condition stabilized over the next 72h. The cyanosis in the palms and in the toes of the left foot receded. The condition of the left toes improved – color and sensation returning to them [Figure 3]. Line of demarcation of the gangrenous area appeared to involve finger tips [Figure 4] and right toes. The patient, after improvement, was shifted to a government hospital due to financial constraints.

## DISCUSSION

Symmetric peripheral gangrene (SPG) is defined as symmetrical distal ischemic damage in two or more sites in the absence of a major vascular occlusive disease. It carries a high mortality rate with a very high frequency of multiple limb amputations in the survivors. A more or less stereotyped clinical picture of SPG in spite of the ever-widening etiological spectrum is suggestive of DIC as the final common pathway of its pathogenesis. It is proposed to be a cutaneous marker of the same.[1]

SPG may manifest unpredictably in conditions associated with sepsis, low output states, vasospastic conditions, myeloproliferative disorders and hyperviscosity syndrome. It has previously been associated with viral gastroenteritis and falciparum malaria.[2,3] Multiple factors like viral gastroenteritis, DIC, dehydration, hypotension and noradrenaline infusion may be implicated in our case. Although gram positive and, less commonly, gram negative bacteria are associated with SPG,[4] in our case, blood, sputum and urine cultures were all negative. Prior antibiotic usage may be an explanation. Recent literature points to a 100% association with DIC, high mortality rate of up to 35%, rates of amputation ranging from 70 to 90% and a possible association with the winter season.[4,5]

The condition is aggravated by asplenism, hypothermia, vasopressor infusion, immunosuppression, diabetes mellitus and renal failure. It may occur as a complication of malignancy, ergot poisoning and increased sympathetic tone states.

The pathogenesis of SPG may include the Schwartzman reaction, bacterial endotoxin release and platelet plugging in peripheral arterioles due to vascular collapse and DIC. Larger vessels are spared and peripheral pulses are generally palpable. Low flow states exacerbate the situation. Discontinuation of vasopressors, reversal of sepsis and DIC and anticoagulation are the suggested first-line measures. Treatment success has been reported for individual patients who received epoprostenol and tissue plasminogen activator infusion, sympathetic blockade, the combination of plasmapheresis, leukapheresis and antibiotics and anticoagulation with heparin and aspirin.[6] Amputation of the affected area may be undertaken once demarcation develops and the patient is stable. At times, spontaneous dropping off is seen.

One case of medicolegal complication is known by the authors and informed consent of relatives is now a protocol in our institute as soon as the first signs of coldness, pallor, dusky hue or pain in peripheries appear or if the patient is referred to us from outside with the same in a patient with risk factors for developing SPG.

## CONCLUSION

SPG carries a high morbidity and mortality. A high index of suspicion and prompt management with usual measures may limit the progression and damage of gangrene, as in our case. The medicolegal aspects of associated gangrene and amputation must be borne in mind.
